# Postoperative complications after salvage mastectomy and repeat breast-conserving surgery in patients with IBTR after previous breast-conserving surgery: a multicenter, retrospective cohort study

**DOI:** 10.1007/s10549-026-07908-6

**Published:** 2026-02-04

**Authors:** Laura M. Tiels, Coco J. E. F. Walstra, Adri C. Voogd, Maurice J. C. van der Sangen, Emiel L. W. G. van Haren, Marjolein L. Smidt, Grard A. P. Nieuwenhuijzen, Robert-Jan Schipper

**Affiliations:** 1 Department of Surgery, Catharina Hospital, Eindhoven, The Netherlands; 2 Department of Epidemiology, Maastricht UMC+, Maastricht, The Netherlands; 3 Department of Research, Netherlands Comprehensive Cancer Organization (IKNL), Utrecht, The Netherlands; 4 Department of Radiotherapy, Catharina Hospital, Eindhoven, The Netherlands; 5 Department of Surgery, Maastricht UMC+, Maastricht, The Netherlands; 6 GROW-School for Oncology and Developmental Biology, Maastricht UMC+, Maastricht, The Netherlands; 7 Department of Plastic- and Reconstructive Surgery, Catharina Hospital, Eindhoven, The Netherlands

**Keywords:** IBTR, Salvage mastectomy, Complications

## Abstract

**Background:**

In patients with ipsilateral breast tumor recurrence (IBTR) previously treated with breast-conserving surgery (BCS) followed by radiotherapy, salvage mastectomy (SM) is still considered standard of care. Currently, there is little evidence available about complication rates of repeat BCS or salvage mastectomy in patients with IBTR and possible differences.

**Aim:**

The primary aim was to report postoperative complication rates after IBTR treatment with salvage mastectomy or repeat BCS after previous BCS (± radiotherapy). Secondary, risk factors associated with complications were examined.

**Methods:**

Complication rates were reported using descriptive statistics. Complications were classified between short-term (less than 3 months after surgery) and long-term (more than 3 months after surgery). Logistic regression was used to evaluate possible risk factors after salvage mastectomy to report an odds ratio (OR) with a 95% confidence interval (CI).

**Results:**

A total of 549 cases with IBTR after primary BCS were included. Short-term complications occurred in 200 (45.2%) of 442 patients treated with salvage mastectomy and in 9 (16.4%) of 55 patients treated with repeat BCS. Seroma and surgical site infection (SSI) were most common in salvage mastectomy (31.7% and 10.9%, respectively). Long-term complications were reported in 16.7% treated with salvage mastectomy and in 14.5% with repeat BCS. The risk of short-term postoperative complications after salvage mastectomy increased significantly with higher BMI. The regression analysis showed that adjuvant radiotherapy after IBTR surgery was associated with long-term postoperative complications.

**Conclusions:**

Salvage mastectomy in case of IBTR after primary BCS is associated with high short-term complication rates, especially seroma. The risk of short-term complications after salvage mastectomy increased with increasing BMI, while adjuvant radiotherapy after salvage mastectomy is associated with long-term complications.

**Supplementary Information:**

The online version contains supplementary material available at 10.1007/s10549-026-07908-6.

## Introduction

In patients with ipsilateral breast tumor recurrence (IBTR) initially treated with breast-conserving surgery (BCS) followed by radiotherapy, salvage mastectomy is still considered standard of care. Nonetheless, a second attempt to preserve the breast, sometimes followed by partial re-irradiation of the breast, is suggested as an oncological safe alternative in selected patients [1].

Currently, little evidence is available about complication rates of second breast-conserving procedure and salvage mastectomy in patients with IBTR. One might expect higher complication rates than after breast conserving treatment (BCT) of the primary tumor since patients were already treated with radiotherapy [2, 3]. Studies have shown that complications after breast surgery result in a reduced quality of life for patients [4–6]. Mastectomy has also been shown to result in a larger reduction of quality of life in comparison to BCT for primary breast cancer [4, 6, 7].

Frequently reported postoperative complications following breast cancer surgery for IBTR (both repeat BCS and mastectomy) include wound infections, seroma, hematoma, skin flap or fat necrosis, and persistent breast pain [5, 8, 9]. Major complications (defined as Clavien–Dindo grade III–V) are reported in 5.9% of patients undergoing breast surgery, and the occurrence of any complication is reported in 36.1% of the cases by the GlobalSurg Collaborative [10]. Numerous studies indicate that postoperative complications occur more frequently after mastectomy than after BCS [11–13].

Several risk factors for complications after surgical treatment of IBTR have been reported. For surgical site infections (SSIs), these include increased age, hypertension, obesity, diabetes, and smoking [14, 15]. Additionally, a previous breast operation has been found to be a significant risk factor [15]. For primary mastectomy, there is evidence that the closing technique influences the occurrence and resolution of postoperative seroma [16, 17].

The primary aim of this study was to examine the postoperative complication rates for IBTR treatment with salvage mastectomy and repeat BCS, after previous BCS (± radiotherapy) for primary breast cancer. The secondary aim was to examine the factors associated with short (< 3 months) and long-term (> 3 months) complication rates.

## Materials and methods

This study is reported according to the Strengthening the Reporting of Observational Studies in Epidemiology (STROBE) guidelines [18]. The Medical research Ethics Committees United (MEC-U) issued a waiver for ethical approval under number W18.148.

### Study design and research population

The study design is a multicenter retrospective cohort study. For inclusion, patients needed to have a history of primary breast cancer treated with BCS, and surgical treatment for IBTR, consisting of either salvage mastectomy or repeat BCS. The omission of surgery for IBTR was an exclusion criterion. A search query through the Dutch National Pathology Archive (PALGA) initially yielded 4,291 cases of possible IBTR diagnosed in 2016 and 2017. Manual screening and elimination of non-applicable cases resulted in a total of 1,494 patients eligible for this study. These patients were treated in 72 hospitals, 34 of which participated in the present study. Local pathology labs were then contacted to find a local treating physician requesting their assistance in providing clinical information through electronic case report forms (eCRFs). In total, 549 eCRFs were completed online by local healthcare professionals from the 34 participating medical centers, utilizing all available clinical information, including operation reports, regular and emergency visits, multidisciplinary meeting notes, and histological records.

### Research population

The study population was based on a multi-center cohort, consisting of patient data from 34 Dutch breast cancer care-providing hospitals between 2016 and 2020. A total of 549 patients with IBTR diagnosed in 2016 and 2017 were included in the database and screened for eligibility in this study. For inclusion, patients needed to have a history of primary breast cancer treated with BCS, and surgical treatment for IBTR, consisting of either salvage mastectomy or repeat BCS.

### Definitions of surgical complications

Complications were classified as short-term (less than 3 months after surgery) and long-term (more than 3 months after surgery). No distinction was made between major and minor complications. Evaluated short-term complications were postoperative hemorrhage, hematoma, surgical site infection (SSI), skin flap necrosis, and seroma. Evaluated long-term complications were impaired shoulder mobility, chronic pain, anterior thoracic wall edema, peripheral edema, and mastitis. Detailed descriptions are provided in Table 1.

**Table 1:** Definitions of surgical complications

	Definition
Short-term complication (< 3 months after surgery)	Postoperative hemorrhage–requiring reoperation Hematoma–requiring needle aspiration Surgical site infection–clinical diagnosis, subdivided in three categories: antibiotic treatment, radiologic intervention, or operative treatment Skin flap necrosis–clinical diagnosis, subdivided in two categories: conservative treatment or operative treatment Seroma–clinical diagnosis, subdivided in three categories: conservative treatment, needle aspiration, or operative treatment
Long-term complication (> 3 months after surgery)	Impaired shoulder mobility–requiring physical therapy Chronic pain–requiring chronic pain medication Anterior thoracic wall edema–requiring physical therapy Peripheral edema–requiring physical therapy Mastitis–clinical diagnosis

### Statistical analysis

Descriptive statistics were used to characterize the study population, including the mean and standard deviation (SD), median and range, or count and percentage, as appropriate. The Chi-square or independent T-test was used to report p-values between the salvage mastectomy and repeat BCS treatment groups, as appropriate. Complication rates were reported using count and percentage. Additionally, we evaluated the incidence of short- and long-term complication rates based on the primary tumor and IBTR treatment modility. P-values were calculated using the Chi-square test or Fisher’s exact test, as appropriate. We used uni- and multivariable logistic regression analyses to evaluate potential risk factors and report odds ratio (OR) with 95% confidence interval (CI). As the number of patients with repeat BCS was too small, uni- and multivariable analyses were only performed for the patients undergoing salvage mastectomy. A p-value less than 0.05 was considered statistically significant. Since previous literature indicated that SSIs are specifically associated with certain risk factors after primary mastectomy [14, 15], we evaluated this complication separately using uni- and multivariable logistic regression analysis. Additionally, we evaluated the association between the closing technique and the occurrence of postoperative seroma in salvage mastectomy, as recent literature provided evidence for this in primary mastectomy [16, 17]. All statistical analyses were performed using IBM SPSS Statistics version 28.0 software.

## Results

### Participants

**Fig. 1:**
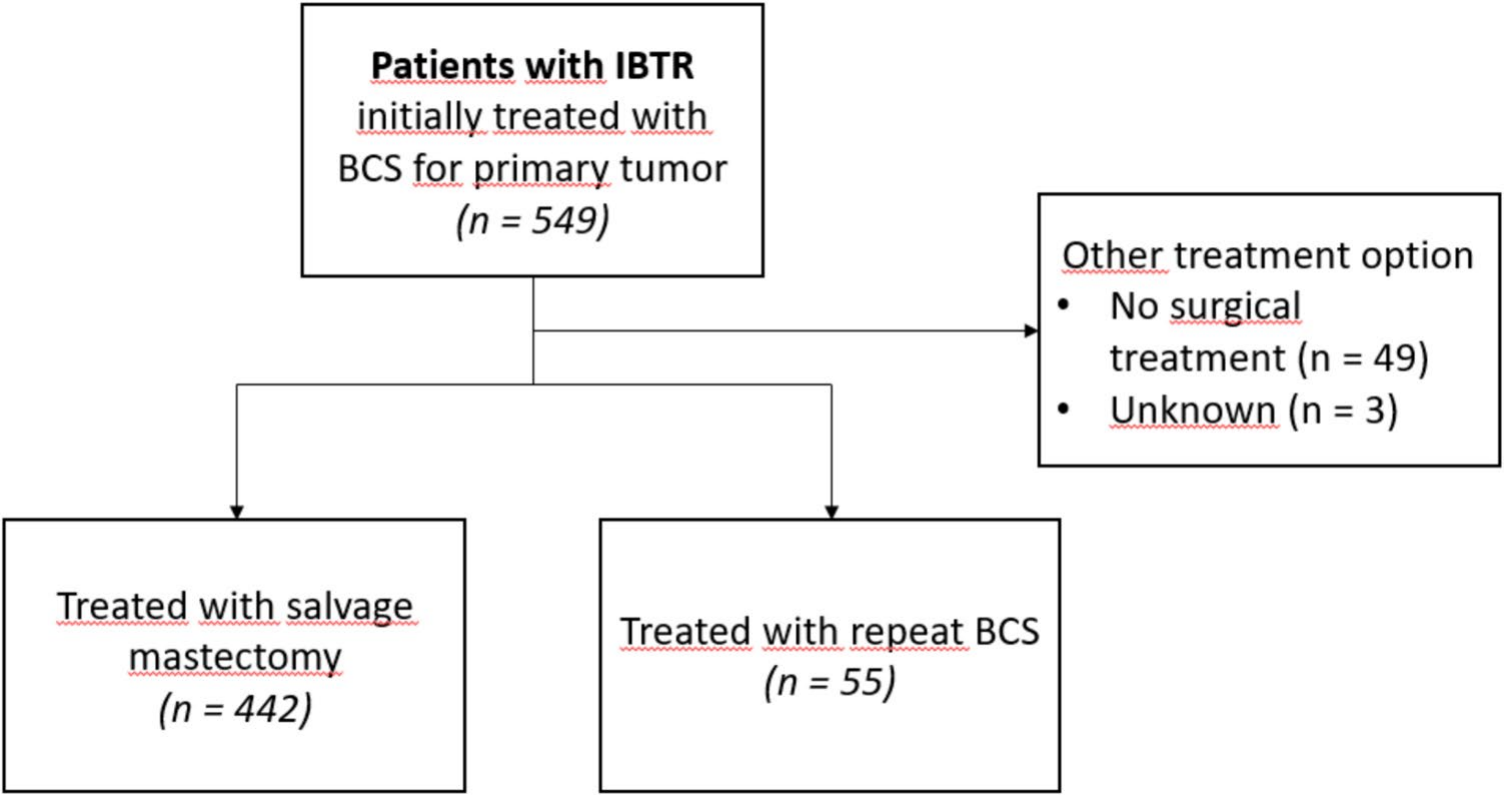
Flowchart for inclusion process. *IBTR* ipsilateral breast tumor recurrence, *BCS* breast-conserving surgery

Among the 549 cases of IBTR after primary BCS (± radiotherapy), 442 (80.5%) were treated with salvage mastectomy, and 55 patients (10.0%) received repeat BCS, and were eligible for inclusion (Fig. 1). Baseline characteristics for the study population per treatment group are shown in Table 2.

**Table 2:** Baseline characteristics

	SM *(N* = *442)*	rBCS *(N* = *55)*	p-value
Age, years (range)	74 (31–96)	76 (53–101)	0.024
BMI, kg/m^2^ (range)	26.3 (16.5–44.3)	24.0 (18.8–43.1)	0.426
Diabetes mellitus Yes No	48 (10.9%) 394 (89.1%)	5 (9.1%) 50 (90.9%)	0.708
Smoking status Former smoker Current smoker Never Unknown	52 (11.8%) 45 (10.2%) 260 (58.8%) 85 (19.2%)	6 (10.9%) 3 (5.5%) 36 (65.5%) 10 (18.2%)	0.652
Radiotherapy for primary breast tumor Yes No Unknown	412 (93.2%) 23 (5.2%) 7 (1.6%)	32 (58.2%) 23 (41.8%)	
DFI < 3 years 3–5 years > 5 years	81 (18.3%) 39 (8.8%) 322 (72.9%)	23 (41.8%) 4 (7.3%) 28 (50.9%)	< 0.001
Tumour grade (Bloom & Richardson) I II III Unknown	68 (15.4%) 207 (46.8%) 130 (29.4%) 37 (8.4%)	12 (21.8%) 22 (40.0%) 15 (27.3%) 6 (10.9%)	0.086
Tumour type IDC ILC Other Unknown	339 (76.6%) 47 (10.6%) 52 (11.9%) 4 (0.9%)	39 (70.9%) 7 (12.7%) 8 (14.5%) 1 (1.8%)	0.847
ER status Positive Negative Unknown	312 (70.6%) 73 (16.5%) 57 (12.9%)	35 (63.6%) 5 (9.1%) 15 (27.3%)	0.982
PR status Positive Negative Unknown	229 (51.8%) 153 (34.6%) 60 (13.6%)	22 (40.0%) 18 (32.7%) 15 (27.3%)	0.713
HER2 status Positive Negative Unknown	42 (9.5%) 336 (76.0%) 64 (14.5%)	6 (10.9%) 32 (58.2%) 17 (30.9%)	0.440
Wound closing technique Conventional closure Flap fixation with subcutaneous sutures (quilting) Flap fixation using tissue adhesive or hemostatic material Other Unknown	372 (84.2%) 24 (5.4%) 2 (0.5%) 30 (6.8%) 14 (3.2%)	N/A N/A N/A N/A N/A	
Postoperative drain Yes No Unknown	337 (76.2%) 88 (29.9%) 17 (3.8%)	4 (7.3%) 44 (80.0%) 7 (12.7%)	< 0.001
Breast reconstruction Yes No Unknown	87 (19.7%) 355 (80.3%) 0 (0.0%)	5 (9.1%) 49 (89.1%) 1 (1.8%)	
Reconstruction type Tissue expander Direct-to-implant prothesis Autologous free-flap Autologous pedicled	33 (7.5%) 8 (1.8%) 28 (6.3%) 23 (5.2%)	2 (3.6%) 0 (0.0%) 0 (0.0%) 3 (5.5%)	
Need for re-excision (involved margins)	12 (2.7%)	10 (18.2%)	
Adjuvant radiotherapy after surgical treatment of the IBTR	58 (13.1%)	19 (34.5%)	

*SM* salvage mastectomy, *rBCS* repeat breast-conserving surgery, *BMI* body mass index, *DFI* disease free interval, *IDC* invasive ductal carcinoma, *ILC* invasive lobular carcinoma, *ER* estrogen receptor, *PR* progesterone receptor, *SN* sentinel node, *ALND* axillary lymph node dissection

### Short-term complications after salvage mastectomy and repeat BCS

In patients treated with salvage mastectomy for IBTR, 200 (45.2%) developed short-term complications. The most frequently reported short-term complications after salvage mastectomy included seroma (31.7%), surgical site infection (SSI) (10.9%), hematoma (3.2%), postoperative hemorrhage (2.7%), and skin flap necrosis (2.3%) (Table 3). Among the patients who developed seroma, 25.9% were treated conservatively, 62.6% required needle aspiration, and 11.5% needed reoperation. Seroma occurred less frequently after conventional wound closure compared to patients where the quilting technique was used (32.5% vs 45.8%, respectively, p = 0.18). SSI was treated with antibiotics in the majority of cases (59.6%). The remaining 40.4% required reoperation. Other reported short-term complications (4.8%) after salvage mastectomy included wound dehiscence and reconstruction complications. In total, 51 patients (11.5%) required re-operation for a short-term complication. Overall, 242 patients (54.8%) did not develop any short-term complications and 38 patients (8.6%) developed two or more.

**Table 3:** Short-term complication rates

	SM *(N* = *442)*	rBCS *(N* = *55)*
Seroma	140 (31.7%)	3 (5.5%)
Surgical site infection	48 (10.9%)	5 (9.1%)
Postoperative hemorrhage	12 (2.7%)	0 (0.0%)
Hematoma	14 (3.2%)	0 (0.0%)
Skin flap necrosis	10 (2.3%)	0 (0.0%)
Other	21 (4.8%)	1 (1.8%)

*SM* salvage mastectomy, *rBCS* repeat breast-conserving surgery

In participants treated with repeat BCS, 9 patients (16.4%) developed short-term complications. Reported short-term complications after repeat BCS were SSI (9.1%) and seroma (5.5%). In total, no patients required re-operation for a short-term complication. Finally, 46 patients (83.6%) did not develop any short-term complications and none developed two or more.

**Fig. 2:**
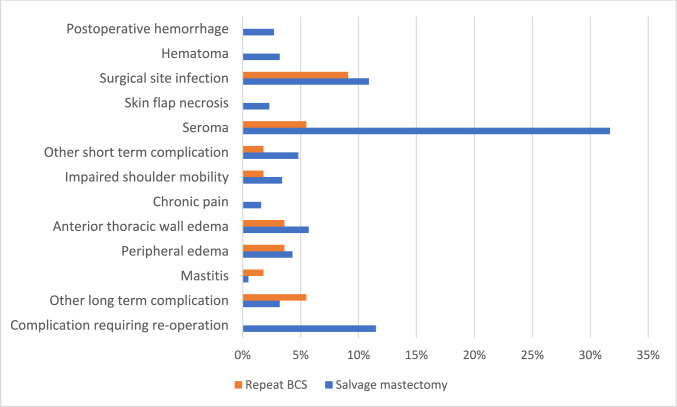
Short- and long-term complication rates after IBTR treatment. *BCS* breast-conserving surgery

Occurrence of any short-term complication stratified by primary tumor and IBTR surgical treatment was shown in Table 4. There was no statistically significant difference in the incidence of short-term complications between primary BCT and primary BCS (43.2% vs 30.4%, respectively, p = 0.22). Among patients who were initially treated with BCS (n = 46), 56.5% (13/23) of those treated with salvage mastectomy for IBTR experienced short-term complications, while 4.3% (1/23) of those treated with BCS experienced short-term complications (p < 0.001). For patients who were initially treated with BCT, including both BCS and radiotherapy (n = 444), the incidence of short-term complications following salvage mastectomy was 44.7% (184/412), whereas 25.0% (8/32) of those treated with repeat BCS had short-term complications (p = 0.04). In the salvage mastectomy group, there was no significant association between prior radiotherapy for the primary tumor and the occurrence of short-term complications (p = 0.51). This was also the case for the repeat BCS group (p = 0.06). Prior radiotherapy treatment was unknown for 7 patients in the salvage mastectomy treatment group, of which 4 patients did not have any short-term complication (Fig. 2).

**Table 4:** Long-term complication rates

	SM *(N* = *442)*	rBCS *(N* = *55)*
Impaired shoulder mobility	15 (3.4%)	1 (1.8%)
Chronic pain	7 (1.6%)	0 (0.0%)
Anterior thoracic wall edema	25 (5.7%)	2 (3.6%)
Peripheral edema	19 (4.3%)	2 (3.6%)
Mastitis	2 (0.5%)	1 (1.8%)
Other	17 (3.8%)	3 (5.5%)

*SM* salvage mastectomy, *rBCS* repeat breast-conserving surgery

### Long-term complications following salvage mastectomy and repeat BCS

Long-term complications following salvage mastectomy were reported in 74 patients (16.7%). The most commonly reported long-term complication was edema of the anterior thoracic wall (5.7%), followed by peripheral edema (4.3%), impaired shoulder mobility (3.4%), chronic pain (1.6%), and mastitis (0.5%) (Table 5). Other reported long-term complications were mainly related to the reconstruction. Overall, 368 patients (83.3%) did not develop any long-term complication. Ten patients (2.2%) developed two or more.

**Table 5:** Short-term complications stratified by primary tumor and IBTR surgical treatment

		Surgical treatment IBTR			
		SM	BCS	Total	p-value
Surgical treatment primary tumor*	BCS	13/23 (56.5%)	1/23 (4.3%)	14/46 (30.4%)	0.04
	BCT (BCS and radiotherapy)	184/412 (44.7%)	8/32 (25.0%)	192/444 (43.2%)	< 0.01
	Total	197/435 (45.3%)	9/55 (16.4%)		

*IBTR* breast tumor recurrence, *SM* salvage mastectomy, *BSC* breast conserving surgery, *BCT* breast conserving therapy

^*^Radiotherapy treatment for primary tumor was unknown for 7 patients in the SM treatment group, of which 4 did not have a short-term complication

Long-term complications after repeat BCS were reported in 8 patients (14.5%). Reported long-term complications included anterior thoracic wall edema (3.6%), peripheral edema (3.6%), impaired shoulder mobility (1.8%), and mastitis (1.8%). In total, 47 patients (85.5%) did not develop any long-term complication and one (1.8%) developed two or more.

Occurrence of any long-term complication stratified by primary tumor and IBTR surgical treatment was shown in Table 6. No statistically significant difference was found in the incidence of long-term complications between primary BCT and primary BCS (16.9% vs 9.4%, respectively p = 0.33). In patients who were initially treated with BCS (n = 53), the incidence of long-term complications following salvage mastectomy was 10.5% (2/19), while the rate was 25.0% (1/4) in the group that received salvage mastectomy with radiotherapy (p = 0.45). For those treated with repeat BCS for IBTR, 10.0% (1/10) experienced long-term complications, and the incidence was 7.7% (1/13) for patients undergoing BCT for IBTR (p = 1.0). For patients initially treated with BCT (n = 443), the incidence of long-term comlications following salvage mastectomy was 15.1% (54/358). This was significantly higher in those receiving salvage mastectomy with radiotherapy, where 27.8% (15/54) developed long-term complications (p = 0.01). Long-term complications following BCS for IBTR occurred in 24.0% (6/25) of cases, while the rate for BCT for IBTR was 0.0% (0/7) (p = 0.71). Radiotherapy treatment for the primary tumor was not statistically associated with long-term complications for both the salvage mastectomy (p = 0.53) and the repeat BCS group (p = 0.45). Prior radiotherapy treatment was unknown for 7 patients in the salvage mastectomy treatment group, of which 5 patients did not have any long-term complication.

**Table 6:** Long-term complications stratified by primary tumor and IBTR surgical treatment

		Surgical treatment IBTR				
		SM	SM and radiotherapy	BCS	BCT	Total
Surgical treatment primary tumor*	BCS	2/19 (10.5%)	1/4 (25.0%)	1/10 (10.0%)	1/13 (7.7%)	5/53 (9.4%)
	BCT (BCS and radiotherapy)	54/358 (15.1%)	15/54 (27.8%)	6/25 (24.0%)	0/7 (0.0%)	75/443 (16.9%)
	Total	56/377 (14.9%)	16/58 (27.6%)	7/35 (20.0%)	1/20 (5.0%)	

*IBTR* breast tumor recurrence, *SM* salvage mastectomy, *BSC* breast conserving surgery, *BCT* breast conserving therapy

*Radiotherapy treatment for primary tumor was unknown for 7 patients in the SM treatment group, of which 5 patients did not have a long-term complication

### Variables associated with higher complication rates

The risk of short-term postoperative complications was significantly associated with age (OR: 1.03; 95% CI 1.01–1.04; p = 0.007), BMI (OR: 1.06; 95% CI 1.02–1.11; p = 0.004), diabetes mellitus (OR: 1.98; 95% CI 1.09–3.66; p = 0.03) and the use of a tissue expander as recontruction type (OR: 0.43; 95% CI 0.19–0.94; p = 0.04). Smoking status, radiotherapy before IBTR surgery, disease free interval (DFI), reconstruction, re-excision, the use of a postoperative drain, and wound closure techniques were not associated with short-term complication risk. The multivariable analysis revealed a significantly higher risk of short-term complications with increasing BMI (OR: 1.05; 95% CI 1.01–1.09; p = 0.02) (Table 7). When looking at SSIs separately as an outcome, only patients undergoing breast reconstruction appeared to have a higher incidence of SSI (OR: 2.54; 95% CI 1.33–4.84; p = 0.005) (Supplemental Table 1). When also taking the type of reconstruction into account, only the autologous free-flap type was found to be significantly associated with SSI (OR: 3.03; 95% CI 1.22–7.57; p = 0.02). Adjuvant radiotherapy after IBTR surgery was associated with long-term postoperative complications (OR: 2.14; 95% CI 1.13–4.06; p = 0.02) (Table 8).

**Table 7:** Uni- and multivariable regression analysis of risk factors associated with any short-term complication

	SM			
	Univariable analysis		Multivariable analysis	
	OR (95% CI)	p-value	OR (95% CI)	p-value
Age, per year increase	1.03 (1.01–1.04)	0.007	1.01 (0.99–1.03)	0.18
Age, categorized Age < 50 Age 50–74 Age 75 +	1 (Reference) 2.22 (0.60–8.30) 3.64 (0.97–13.60)	0.24 0.06		
BMI, per point increase	1.06 (1.02–1.11)	0.004	1.05 (1.01–1.09)	0.02
BMI, categorized BMI < 25.00 BMI 25.00–29.99 BMI > 30.00	1 (Reference) 2.53 (1.61–3.97) 2.02 (1.23–3.34)	< 0.001 0.006		
Diabetes mellitus (yes vs. no)	1.98 (1.08–3.66)	0.03	1.52 (0.81–2.86)	0.20
Smoking status Never Current smoker Former smoker	1 (Reference) 0.80 (0.42–1.53) 1.40 (0.77–2.55)	0.50 0.27		
Radiotherapy before IBTR surgery (yes vs. no)	0.71 (0.34–1.49)	0.36		
DFI < 3 years 3–5 years > 5 years	1 (Reference) 1.39 (0.67–2.83) 1.33 (0.85–2.09)	0.39 0.21		
Postoperative drain (yes vs. no)	0.65 (0.41–1.05)	0.08		
Reconstruction (yes vs. no)	0.73 (0.45–1.18)	0.20		
Reconstruction type Tissue expander (yes vs. no) Direct-to-implant prothesis (yes vs. no) Autologous free-flap (yes vs. no) Autologous pedicled (yes vs. no)	0.43 (0.19–0.94) 0.72 (0.17–3.06) 1.23 (0.57–2.64) 1.12 (0.48–2.59)	0.04 0.66 0.60 0.80	0.42 (0.17–1.03)	0.06
Reexcision (yes vs. no)	0.60 (0.18–2.01)	0.41		
Wound closure technique Conventional closure Flap fixation with subcutaneous sutures (quilting)	1 (Reference) 1.45 (0.63–3.32)	0.38		

*SM* salvage mastectomy, *OR* odds ratio, *CI* confidence interval, *BMI* body mass index, *IBTR* ipsilateral breast tumor recurrence, *DFI* disease free interval

**Table 8:** Univariable regression analysis of variables associated with any long-term complication

	SM	
	OR (95% CI)	p-value
Age, per point increase	1.00 (0.98–1.02)	0.95
BMI, per point increase	1.03 (0.98–1.08)	0.32
Diabetes mellitus (yes vs. no)	0.69 (0.28–1.69)	0.42
Smoking status		
Never	1 (Reference)	
Current smoker	0.30 (0.09–1.01)	0.05
Former smoker	0.65 (0.28–1.54)	0.33
Radiotherapy before IBTR surgery (yes vs. no)	1.01 (0.37–2.72)	0.99
Postoperative drain (yes vs. no)	0.82 (0.45–1.49)	0.51
Reconstruction (yes vs. no)	1.52 (0.85–2.73)	0.16
Reconstruction type		
Tissue expander (yes vs. no)	1.11 (0.44–2.80)	0.82
Direct-to-implant prothesis (yes vs. no)	1.68 (0.33–8.47)	0.53
Autologous free-flap (yes vs. no)	2.54 (1.10–5.87)	0.03
Autologous pedicled (yes vs. no)	0.46 (0.11–2.00)	0.46
Reexcision (yes vs. no)	0.45 (0.06–3.50)	0.44
Wound closure technique Conventional closure Flap fixation with subcutaneous sutures (quilting)	1 (Reference) 0.46 (0.10–1.98)	0.29
Radiotherapy after IBTR surgery (yes vs. no)	2.14 (1.13–4.06)	0.02

*SM* salvage mastectomy, *OR* odds ratio, *CI* confidence interval, *BMI* body mass index, *IBTR* ipsilateral breast tumor recurrence

## Discussion

This multicenter cohort study presents the short- and long-term complication rates for salvage mastectomy and repeat BCS for IBTR. Short-term complications were observed in 45.2% of patients undergoing salvage mastectomy compared to 16.4% of those receiving repeat BCS, mostly consisting of seroma formation. Long-term complications occurred in 16.7% of patients who had salvage mastectomy and 14.5% of those treated with repeat BCS. In the salvage mastectomy group, short-term complications were linked to factors such as age, BMI, diabetes mellitus, and the use of a tissue expander for breast reconstruction. Furthermore, autologous free-flap reconstruction was associated with a higher incidence of surgical site infections (SSI) in the salvage mastectomy cohort. Adjuvant radiotherapy after IBTR surgery was associated with long-term postoperative complications.

Our analysis revealed noteworthy differences in the indidence of short- and long-term complication rates based on the primary tumor treatment modility. Overall, there was no statistically significant difference in the incidence of short-term complications between primary BCT and primary BCS (43.2% vs 30.4%, respectively, p = 0.22). For long-term complications, no statistically significant difference was found in the incidence of long-term complications between primary BCT and primary BCS (16.9% vs 9.4%, respectively p = 0.33). These findings suggest that the primary treatment modality may influence the complication rates following IBTR treatment, although both findings were not statistically significant and therefore further studies with larger sample sizes may be needed to confirm this finding. Also, in our uni- and multivariable analysis for the salvage mastectomy group, radiotherapy before IBTR surgery was not found to be a statistically significant factor associated with higher risk of any short- or long-term complication. Therefore, the clinical impact of these results remains unclear, even though emphasizing the need for careful consideration when choosing IBTR surgical treatment.

Patients who initially underwent BCS and later salvage mastectomy had a lower incidence of long-term complications (10.5%) compared to those who underwent salvage mastectomy combined with radiotherapy (25.0%), but this difference was not statistically significant (p = 0.45). In contrast, for patients with primary BCT, the incidence of long-term complications following salvage mastectomy was significantly higher in those receiving radiotherapy (27.8%) compared to those who did not receive radiotherapy (15.1%) (p = 0.01). These findings suggest that salvage mastectomy with radiotherapy may carry an increased risk of long-term complications, particularly in patients who previously received radiotherapy as part of their primary tumor treatment, which was also confirmed in our regression analysis. Together, these findings highlight the importance and need for a tailored approach to minimize complications and optimize outcomes for patients with IBTR.

The present study reported a short-term complication rate of 45.2%. For salvage mastectomy following previous BCS (± radiotherapy), the available data on complication rates are limited. Only one article involving a similar patient population with IBTR reported a 30-day postoperative complication rate of 20% [19]. The most common complication in this group was an SSI, which occurred in 8% of patients, followed by seroma (5%), hematoma (4%), and skin necrosis (3%). Our study population experienced higher postoperative morbidity compared to the previously mentioned study. We attribute this to the higher risk of seroma in our cohort. The additional complication rates aligned with our study findings. It is important to note that the aforementioned study did not investigate long-term complications. Additionally, the definitions of complications differed between the studies. Nevertheless, the risks of complications following salvage mastectomy remain significant.

In the present study, we found a short-term complication rate of 16.4% after repeat BCS. Data on postoperative complications following repeat lumpectomy and subsequent radiotherapy are also limited. One single-centre study has reported a 30-day postoperative complication rate of 6% in a relatively small population (n = 113), as opposed to 20% in salvage mastectomy [20]. Less than half of the repeat lumpectomy patients (41%) had re-irradiation. The RTOG 1014-group reported up to 25% of low-grade adverse events, most of which were irradiation-related, in the first year after repeat lumpectomy followed by reirradiation in a series of 58 patients [21]. In this study, only 9% of patients developed seroma. One might suggest that complications after repeat BCS are less common compared to salvage mastectomy.

Several studies have reported on 30-day postoperative morbidity following primary breast surgery. The GlobalSurg Collaborative indicated that major complications (defined as Clavien–Dindo grade III–V) occurred in 5.9% of patients undergoing breast surgery, and any complication in 28.7–36.1% [10]. Other studies reported major postoperative complications varying from 4.0 to 7.3% for mastectomy and from 1.6% to 2.3% for lumpectomy [11–13, 22, 23]. Our study shows a slightly higher incidence of any postoperative complications, both for salvage mastectomy and repeat BCS for IBTR. This difference can likely be attributed to previous surgery and the fact that most patients underwent radiation therapy after primary BCS.

In primary mastectomy, the closure technique appears to affect the risk of seroma formation [16, 17]. In our study, the quilting technique was used in only 24 patients instead of conventional closure in 372 patients. There was no significant difference in the incidence of any early or late postoperative complications in the analysis. The only trend observed after statistical analysis was towards a higher incidence of SSIs after quilting technique (p = 0.06, see Supplemental Table 1). Based on these limited and unbalanced data, no conclusion can be made about the additional value of quilting in salvage mastectomy. Further research on this topic is needed.

We analyzed risk factors for SSIs separately because more prior research is available on this specific complication. The reported SSI incidence in previous findings varied from 2.1 to 7.8% despite the treatment choice [24, 25]. When differentiating between both treatment options, lumpectomy showed an SSI rate of 1.3–2.0%, while mastectomy showed an SSI rate of 2.3% to 4.3% [13, 22–24, 26]. Our complication rates were slightly elevated compared to other study populations. However, the overall trend was similar. Studies showed that multiple factors, including high BMI, diabetes, and smoking were associated with a higher risk of SSIs [15, 22, 24]. Studies reporting on age as a risk factor are contradictory [27, 28]. A recent randomized controlled trial found that only a BMI of 30 or higher was significantly associated with SSI [29]. Our analysis showed that only breast reconstruction after salvage mastectomy was associated with a higher risk of SSI (p = 0.01). When looking at the reconstruction type, only the autologous free-flap was found to significantly increase the risk of SSI. A recent systematic review and meta-analysis showed that a particular reconstruction type did not increase complication rates [30]. For patients treated with salvage mastectomy after previous BCS (± radiotherapy), complication rates were also not associated with the reconstruction type [31].

The retrospective design of the present study is a limitation since some complications could be missed during data registration and no distinction could be made between minor and major complications. Nonetheless, this is a unique multicenter study presenting current daily practice. Since we included IBTR patients of multiple centers in the Netherlands treated with salvage mastectomy or repeat BCS for a period of two years, we believe the study population is therefore a representative sample of the current population of patients diagnosed with IBTR. However, the study size of patients treated with repeat BCS was relatively small since salvage mastectomy is still the standard treatment in IBTR patients in the current surgical practice. Future research is required to examine further postoperative complication rates, as well as associations with possible risk factors.

In conclusion, breast surgery for IBTR has low rates of surgical morbidity, akin to those seen in primary breast surgery. Patients undergoing salvage mastectomy appear to experience high complication rates, especially seroma. Furthermore, short-term complications were linked to higher BMI, while adjuvant radiotherapy after salvage mastectomy is associated with long-term complications.

## Supplementary Information

Below is the link to the electronic supplementary material.

Supplementary file1 (DOCX 13 kb)

## Data Availability

The datasets generated during and/or analysed during the current study are not publicly available but are available from the corresponding author on reasonable request.

## References

[1] Walstra C, Schipper RJ, Poodt IGM, van Riet YE, Voogd AC, van der Sangen MJC, Nieuwenhuijzen GAP (2019) Repeat breast-conserving therapy for ipsilateral breast cancer recurrence: a systematic review. Eur J Surg Oncol 45(8):1317–1327 30795956 10.1016/j.ejso.2019.02.008

[2] Kanda MH, da Costa Vieira RA, Lima J, Paiva CE, de Araujo RLC (2020) Late locoregional complications associated with adjuvant radiotherapy in the treatment of breast cancer: systematic review and meta-analysis. J Surg Oncol 121(5):766–776 31879978 10.1002/jso.25820

[3] Elfgen C, Guth U, Gruber G, Birrer S, Bjelic-Radisic V, Fleisch M, Tausch CJ (2020) Breast-conserving surgery with intraoperative radiotherapy in recurrent breast cancer: the patient’s perspective. Breast Cancer 27(6):1107–1113 32488732 10.1007/s12282-020-01114-y PMC7567708

[4] Kouwenberg CAE, de Ligt KM, Kranenburg LW, Rakhorst H, de Leeuw D, Siesling S et al (2020) Long-term health-related quality of life after four common surgical treatment options for breast cancer and the effect of complications: a retrospective patient-reported survey among 1871 patients. Plast Reconstr Surg 146(1):1–13 32590633 10.1097/PRS.0000000000006887

[5] Thalji SZ, Cortina CS, Guo MS, Kong AL (2023) Postoperative complications from breast and axillary surgery. Surg Clin North Am 103(1):121–139 36410345 10.1016/j.suc.2022.08.007

[6] Enien MA, Ibrahim N, Makar W, Darwish D, Gaber M (2018) Health-related quality of life: impact of surgery and treatment modality in breast cancer. J Cancer Res Ther 14(5):957–963 30197331 10.4103/0973-1482.183214

[7] Rautalin M, Jahkola T, Roine RP (2021) Surgery and health-related quality of life - a prospective follow up study on breast cancer patients in Finland. Eur J Surg Oncol 47(7):1581–1587 33593622 10.1016/j.ejso.2021.02.006

[8] Vitug AF, Newman LA (2007) Complications in breast surgery. Surg Clin North Am 87(2):431–451. 10.1016/j.suc.2007.01.005 17498536 10.1016/j.suc.2007.01.005

[9] Al-Hilli Z, Wilkerson A (2021) Breast surgery: management of postoperative complications following operations for breast cancer. Surg Clin North Am 101(5):845–863 34537147 10.1016/j.suc.2021.06.014

[10] GlobalSurg C, National Institute for Health Research Global Health Research Unit on Global S (2021) Global variation in postoperative mortality and complications after cancer surgery: a multicentre, prospective cohort study in 82 countries. Lancet 397(10272):387–397 33485461 10.1016/S0140-6736(21)00001-5 PMC7846817

[11] de Boniface J, Szulkin R, Johansson ALV (2022) Major surgical postoperative complications and survival in breast cancer: Swedish population-based register study in 57 152 women. Br J Surg 109(10):977–983 35929050 10.1093/bjs/znac275 PMC10364684

[12] Chatterjee A, Pyfer B, Czerniecki B, Rosenkranz K, Tchou J, Fisher C (2015) Early postoperative outcomes in lumpectomy versus simple mastectomy. J Surg Res 198(1):143–148 26070497 10.1016/j.jss.2015.01.054

[13] Pyfer B, Chatterjee A, Chen L, Nigriny J, Czerniecki B, Tchou J, Fisher C (2016) Early postoperative outcomes in breast conservation surgery versus simple mastectomy with implant reconstruction: a NSQIP analysis of 11,645 patients. Ann Surg Oncol 23(1):92–98 26219243 10.1245/s10434-015-4770-2

[14] McNeely ML, Binkley JM, Pusic AL, Campbell KL, Gabram S, Soballe PW (2012) A prospective model of care for breast cancer rehabilitation: postoperative and postreconstructive issues. Cancer 118(8 Suppl):2226–2236 22488697 10.1002/cncr.27468

[15] Xue DQ, Qian C, Yang L, Wang XF (2012) Risk factors for surgical site infections after breast surgery: a systematic review and meta-analysis. Eur J Surg Oncol 38(5):375–381 22421530 10.1016/j.ejso.2012.02.179

[16] ten Wolde B, van den Wildenberg FJ, Keemers-Gels ME, Polat F, Strobbe LJ (2014) Quilting prevents seroma formation following breast cancer surgery: closing the dead space by quilting prevents seroma following axillary lymph node dissection and mastectomy. Ann Surg Oncol 21(3):802–807 24217790 10.1245/s10434-013-3359-x

[17] van SpiekermanWeezelenburg MA, Bakens M, Daemen JHT, Aldenhoven L, van Haaren ERM, Janssen A et al (2024) Prevention of seroma formation and its sequelae after axillary lymph node dissection: An Up-to-Date Systematic Review and Guideline for Surgeons. Ann Surg Oncol 31(3):1643–1652 38038792 10.1245/s10434-023-14631-9

[18] von Elm E, Altman DG, Egger M, Pocock SJ, Gotzsche PC, Vandenbroucke JP, Initiative S (2014) The strengthening the reporting of observational studies in epidemiology (STROBE) statement: guidelines for reporting observational studies. Int J Surg 12(12):1495–1499 25046131 10.1016/j.ijsu.2014.07.013

[19] ElSherif A, Armanyous S, Al-Hilli Z, Valente SA (2022) Mastectomy options for the treatment of ipsilateral breast cancer recurrence after lumpectomy. Am J Surg 223(3):447–451 34955166 10.1016/j.amjsurg.2021.11.028

[20] ElSherif A, Shah C, Downs-Kelly E, Alhareb A, Valente SA, Tu C, Al-Hilli Z (2022) Outcomes of ipsilateral breast tumor recurrence after breast conserving surgery: repeat lumpectomy as an alternative to salvage mastectomy. Surgery 171(3):673–681 34911644 10.1016/j.surg.2021.10.069

[21] Arthur DW, Winter KA, Kuerer HM, Haffty BG, Cuttino LW, Todor DA et al (2017) NRG oncology-radiation therapy oncology group study 1014: 1-year toxicity report from a phase 2 study of repeat breast-preserving surgery and 3-dimensional conformal partial-breast reirradiation for in-breast recurrence. Int J Radiat Oncol Biol Phys 98(5):1028–1035 28721885 10.1016/j.ijrobp.2017.03.016 PMC5572128

[22] de Blacam C, Ogunleye AA, Momoh AO, Colakoglu S, Tobias AM, Sharma R et al (2012) High body mass index and smoking predict morbidity in breast cancer surgery: a multivariate analysis of 26,988 patients from the national surgical quality improvement program database. Ann Surg 255(3):551–555 22330036 10.1097/SLA.0b013e318246c294

[23] El-Tamer MB, Ward BM, Schifftner T, Neumayer L, Khuri S, Henderson W (2007) Morbidity and mortality following breast cancer surgery in women: national benchmarks for standards of care. Ann Surg 245(5):665–671 17457156 10.1097/01.sla.0000245833.48399.9a PMC1877061

[24] Pastoriza J, McNelis J, Parsikia A, Lewis E, Ward M, Marini CP, Castaldi MT (2021) Predictive factors for surgical site infections in patients undergoing surgery for breast carcinoma. Am Surg 87(1):68–76 32927974 10.1177/0003134820949996

[25] Throckmorton AD, Boughey JC, Boostrom SY, Holifield AC, Stobbs MM, Hoskin T et al (2009) Postoperative prophylactic antibiotics and surgical site infection rates in breast surgery patients. Ann Surg Oncol 16(9):2464–2469 19506959 10.1245/s10434-009-0542-1

[26] Davis GB, Peric M, Chan LS, Wong AK, Sener SF (2013) Identifying risk factors for surgical site infections in mastectomy patients using the National Surgical Quality Improvement Program database. Am J Surg 205(2):194–199 22944390 10.1016/j.amjsurg.2012.05.007

[27] de Boniface J, Szulkin R, Johansson ALV (2023) Medical and surgical postoperative complications after breast conservation versus mastectomy in older women with breast cancer: Swedish population-based register study of 34 139 women. Br J Surg 110(3):344–352 36511352 10.1093/bjs/znac411 PMC10364521

[28] Ten Wolde B, Kuiper M, de Wilt JHW, Strobbe LJA (2017) Postoperative complications after breast cancer surgery are not related to age. Ann Surg Oncol 24(7):1861–1867 28168385 10.1245/s10434-016-5726-x

[29] Stallard S, Savioli F, McConnachie A, Norrie J, Dudman K, Morrow ES, Romics L (2022) Antibiotic prophylaxis in breast cancer surgery (PAUS trial): randomised clinical double-blind parallel-group multicentre superiority trial. Br J Surg 109(12):1224–1231 35932230 10.1093/bjs/znac280 PMC10364710

[30] Stefura T, Rusinek J, Wator J, Zagorski A, Zajac M, Libondi G et al (2023) Implant vs. autologous tissue-based breast reconstruction: a systematic review and meta-analysis of the studies comparing surgical approaches in 55,455 patients. J Plast Reconstr Aesthet Surg. 77:346–358 36621238 10.1016/j.bjps.2022.11.044

[31] Asaad M, Mitchell D, Murphy B, Liu J, Selber JC, Clemens MW et al (2023) Surgical outcomes of implant versus autologous breast reconstruction in patients with previous breast-conserving surgery and radiotherapy. Plast Reconstr Surg 151(2):190e-e199 36332081 10.1097/PRS.0000000000009826

