# Supplementary material for: Postoperative complications after salvage mastectomy and repeat breast-conserving surgery in patients with IBTR after previous breast-conserving surgery: a multicenter, retrospective cohort study
Source: Breast Cancer Res Treat. 2026 Feb 4;215(3):71. doi: 10.1007/s10549-026-07908-6 (PMC12872630; doi:10.1007/s10549-026-07908-6)
Supplement: Supplementary file 1 — Supplementary file1 (DOCX 13 kb) [file 10549_2026_7908_MOESM1_ESM.docx]

**SUPPLEMENTAL MATERIALS**

Supplemental Table 1. Univariable regression analysis of variables associated with surgical site infections (SSIs)

|  | SM | |
| --- | --- | --- |
|  | **OR (95% CI)** | **p-value** |
| Age, per point increase | 1.00 (0.97-1.02) | 0.81 |
| BMI, per point increase | 1.04 (0.98-1.11) | 0.20 |
| Diabetes mellitus (yes vs. no) | 0.97 (0.36-2.57) | 0.95 |
| Smoking status |  |  |
| Never | 1 (Reference) |  |
| Current smoker | 0.57 (0.17-1.95) | 0.37 |
| Former smoker | 1.67 (0.74-3.77) | 0.22 |
| Radiotherapy before IBTR surgery (yes vs. no) | 1.10 (0.32-3.78) | 0.88 |
| Postoperative drain (yes vs. no) | 0.93 (0.44-1.96) | 0.86 |
| Reconstruction (yes vs. no) | 2.54 (1.33-4.84) | 0.005 |
| Reconstruction type |  |  |
| Tissue expander (yes vs. no) | 1.94 (0.76-4.97) | 0.17 |
| Direct-to-implant prothesis (yes vs. no) | 2.81 (0.55-14.34) | 0.21 |
| Autologous free-flap (yes vs. no) | 3.03 (1.22-7.57) | 0.02 |
| Autologous pedicled (yes vs. no) | 2.43 (0.86-6.87) | 0.09 |
| Reexcision (yes vs. no) | - | 1.0 |
| Wound closure technique  Conventional closure  Flap fixation with subcutaneous sutures (quilting) | 1 (Reference)  0.39 (0.05-3.00) | 0.37 |

Definitions of abbreviations: SM = salvage mastectomy; OR = odds ratio; CI = confidence interval; BMI = body mass index; IBTR = ipsilateral breast tumor recurrence.
